# Mineralocorticoid Receptor Antagonists and Cognitive Outcomes in Cardiovascular Disease and Beyond: A Systematic Review

**DOI:** 10.3390/jpm15020057

**Published:** 2025-01-30

**Authors:** Paola Pastena, Gabriele Campagnoli, Ali Reza Rahmani, Andreas P. Kalogeropoulos

**Affiliations:** 1 Division of Cardiology, Department of Pediatrics, Columbia University Irving Medical Center, New York, NY 10032, USA; 2 Faculty of Medicine and Surgery, University of Milan, 20122 Milan, Italy; 3 Division of Cardiology, Department of Medicine, Stony Brook University, Stony Brook, NY 11794, USA; 4 Health Sciences Center, Stony Brook University Medical Center, Stony Brook, NY 11794, USA

**Keywords:** cognitive outcomes, mineralocorticoid receptor antagonists, spironolactone, cardiovascular disease

## Abstract

**Background/Objectives:** Cognitive impairment is a debilitating comorbidity affecting diverse patient populations, yet the cognitive effects of therapies like mineralocorticoid receptor antagonists (MRAs) remain underexplored. Preclinical evidence suggests that MRAs, particularly spironolactone, may reduce cognitive decline by modulating aldosterone-dependent pathways and targeting hippocampal receptors. However, evidence in humans is fragmented, and no systematic review has consolidated these findings. This review evaluates the cognitive effects of MRAs, synthesizes current data, and identifies research gaps. **Methods:** A literature search using terms related to MRAs and cognitive outcomes was performed in PubMed and Web of Science from 1979 to 2023. A total of 143 articles were identified and 85 were screened after removing duplicates. Ultimately, 44 studies were included and were classified based on study design and population focus (preclinical, healthy controls, patients with psychiatric disorders, and cardiovascular patients). **Results:** Spironolactone demonstrated mixed effects on cognition. In healthy participants, it improved spatial memory under stress and prevented stress-related suppression of medial temporal activity, but impaired working memory and selective attention. In patients with psychiatric conditions, spironolactone reduced cognitive empathy deficits in major depressive disorder and improved working memory in bipolar I disorder. In cardiovascular patients, spironolactone improved cognitive scores and hippocampal memory but had no effect on non-hippocampal memory. **Conclusions:** Spironolactone exhibits potential cognitive benefits across diverse populations. However, its effects on cognition are mixed, highlighting the need for further research to understand its mechanisms and therapeutic potential, particularly in patients with heart failure and other related conditions.

## 1. Introduction

Cognitive decline is a debilitating condition that affects a significant proportion of the global population, with its prevalence rising due to aging and the growing burden of chronic illnesses, such as cardiovascular diseases [1,2]. The presence of cognitive impairment, even at subclinical levels, has been associated with higher hospitalization rates, poorer quality of life, and increased mortality, making it an independent prognostic factor [1,3].

Despite its significant impact, limited data are available regarding the effectiveness and safety of treatments specifically targeting cognitive impairment, with most existing interventions focusing primarily on managing underlying conditions rather than addressing cognitive decline directly. In cardiac patients with diagnosed cognitive impairment, recommendations emphasize optimal medical management to mitigate shared risk factors. However, these guidelines are largely informed by expert opinion and a lack of robust support from clinical studies [4].

Interestingly, preclinical studies have suggested that spironolactone, a widely used MRA in HF treatment, may mitigate cognitive decline [5,6]. This potential benefit of MRAs is believed to stem from blocking certain effects of aldosterone, as elevated aldosterone levels are associated with an increased risk of cognitive impairment [7]. Additionally, mineralocorticoid receptors (MRs), which are abundant in critical brain regions like the hippocampus, an area essential for memory and highly affected in Alzheimer’s disease, may serve as targets for spironolactone’s cognitive effects [6].

While promising findings from experimental animal studies suggest that MRAs, such as spironolactone, may prevent cognitive decline, evidence in humans remains limited, and the existing literature on this topic is highly fragmented. To our knowledge, no systematic review has yet synthesized the available clinical evidence on whether MRAs can mitigate cognitive decline in humans. Therefore, our review aims to evaluate the current human studies investigating the cognitive effects of MRAs, summarize their main findings, and identify areas requiring further research. By consolidating the existing evidence, we aim to provide insights into the potential role of MRAs as therapeutic agents in reducing cognitive decline.

## 2. Materials and Methods

This systematic review was not registered in a protocol registry; however, the methodology adhered to the guidelines outlined in the Preferred Reporting Items for Systematic Reviews and Meta-Analyses (PRISMA) statement to ensure rigor and transparency [8]. A meta-analysis was not performed due to the heterogeneity of the study populations and the variability in reported outcomes.

### 2.1. Literature Search

To identify studies in the literature exploring the cognitive effects of MRAs, we conducted a comprehensive search across two databases, PubMed and Web of Science, covering the period from 1979 to 2023. The search string applied was as follows: (spironolactone OR “mineralocorticoid receptor antagonist” OR “aldosterone antagonist” OR “MR antagonist” OR “K + sparing diuretic” OR “aldactone”) AND (cognit * OR “cognitive dysfunction” OR “cognitive impairment” OR dementia OR “memory loss” OR “Alzheimer’s disease” OR “neurocognitive disorder”).

### 2.2. Inclusion and Exclusion Criteria

Studies were included if they investigated the cognitive effects of MRAs (e.g., spironolactone, eplerenone) in preclinical or human populations, focusing on outcomes such as memory, learning, executive function, or other neurocognitive measures. We excluded studies that did not directly address cognitive outcomes or were limited to psychiatric conditions without assessing cognitive performance. Papers that exclusively investigated glucocorticoid receptor (GR) antagonists or other unrelated interventions were also excluded. Reviews, conference papers, letters, editorials, book chapters, and articles not published in English language were not considered.

### 2.3. Study Selection and Data Extraction

The articles retrieved from our search were screened using the Rayyan Intelligent Systematic review platform [9]. Following the removal of duplicate entries (assessed based on identical titles, authorship, journal information, or publication details), two authors (P.P. and G.C.) independently assessed the studies by reviewing their titles and abstracts. A subsequent full-text review of the shortlisted articles was conducted by the same authors to determine eligibility. Key details from the selected studies, such as the title, author information, publication year, and DOI, were extracted and systematically recorded in a Microsoft Excel file.

**Figure 1:**
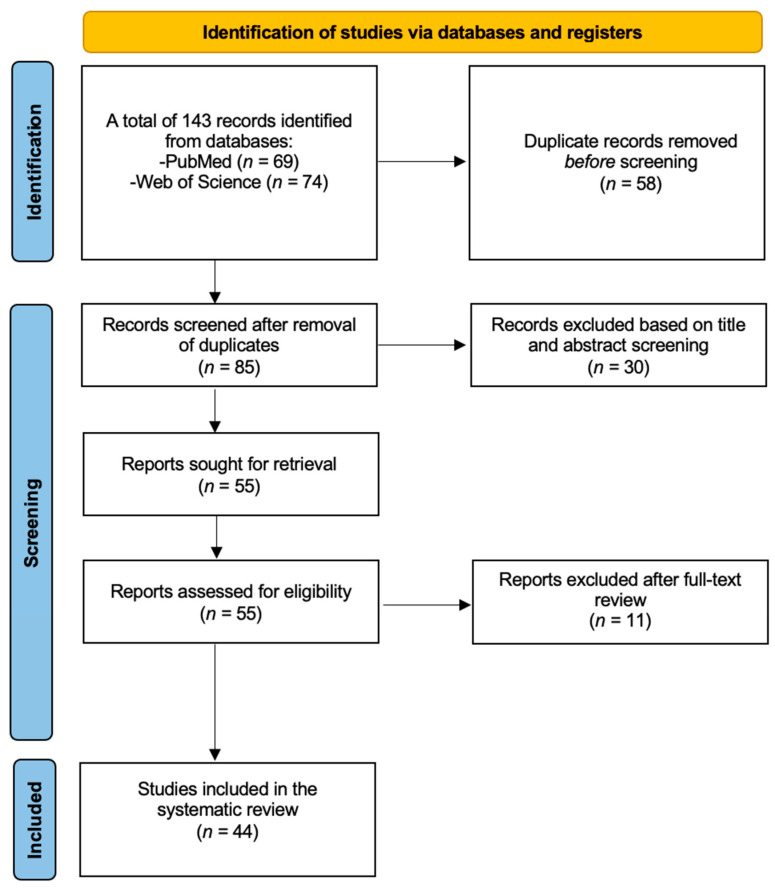
PRISMA flow diagram for the inclusion of studies.

An overview of our search strategy, along with the PRISMA flow diagram, is presented in Figure 1. Our initial search across two databases identified a total of 143 studies, with 69 retrieved from PubMed and 74 from Web of Science. After removing duplicates, 85 articles remained. Of these, 30 studies were excluded after title and abstract screening. The remaining 55 articles underwent full-text review, leading to the exclusion of 11 additional studies. A total of 44 articles were included in our systematic review.

### 2.4. Data Synthesis

The selected papers were divided by study design into two sections: preclinical (28 articles) and clinical studies (16 articles). The clinical studies were further categorized into subgroups based on the study population: healthy volunteers (8 articles), individuals with psychiatric disorders (5 articles), and patients with cardiovascular risk factors (3 articles). For each article, we collected the following information: authors, year of publication, study design, specifics of the population, exposure, outcome, and main findings. Data from included studies were synthesized narratively and presented in tabular format to summarize study characteristics and outcomes.

Heterogeneity among study results was assessed narratively by comparing differences in study populations, intervention characteristics, and outcome measures, while sensitivity analyses were not conducted as the review employed a narrative synthesis without quantitative pooling of data.

### 2.5. Risk of Bias

A formal risk of bias assessment was not conducted for this review due to the significant heterogeneity among the included studies in terms of design, population, intervention, and outcome measures.

## 3. Results

### 3.1. Preclinical Studies

Preclinical studies in animal models extensively investigated the potential effects of MRAs on cognition. Table 1 provides an overview of the included articles, detailing their study designs, specifics of the population, exposure of interest, outcome measures, and main findings.

**Table 1:** Preclinical studies investigating the effect of MRAs on cognition.

Authors	Year	Study Type	Species	Experimental Model	Exposure of Interest	Outcome	Main Findings
Yau et al. [10]	2011	Preclinical study	Mouse/Rat	Aged 11βHSD1 deficient mice and control mice	MRA (spironolactone) and GR antagonist (RU486)	Spatial memory	Spironolactone did not improve cognitive decline. It impaired memory in mice with previously intact spatial memory (11βHSD1 deficient mice) and had no improvement in the control mice with already impaired memory.
Atucha et al. [11]	2015	Preclinical study	Mouse/Rat	5 to 7 rats receiving C118335 and 5 to 7 rats as control receiving vector	Selective GR modulator and MRA (C118335); GR antagonist (RU486); MRA (spironolactone)	Memory consolidation	Spironolactone impaired memory consolidation when administered post-training.
Zhou et al. [12]	2011	Preclinical study	Mouse/Rat	Healthy mice undergoing fear conditioning	MRA spironolactone; GR antagonist RU486	Effects on fear memory retrieval (contextual and tone-cued fear)	Spironolactone reduced the expression of contextual fear memory when administered prior to retrieval but had no effect on tone-cued fear memory.
Smythe et al. [13]	1997	Preclinical study	Mouse/Rat	10 mice per group in experiment 1, 6 mice per group in experiment 2	MRA spironolactone in rats with scopolamine-induced cognitive impairment	Cognitive performance in a water maze task (memory and spatial learning)	Systemic spironolactone improved cognitive performance in rats with scopolamine-induced cognitive impairment
Yau et al. [14]	1999	Preclinical study	Mouse/Rat	Male lister hooded rats divided in 4 groups: MRA, GR antagonist, acute swim stress, control	GR antagonist RU38486/mifepristone, MRA spironolactone, acute swim stress	Spatial learning and memory in water maze	Chronic MR blockade with spironolactone impaired spatial memory retention, decreasing time spent in the target quadrant, suggesting MR activation supports cognitive function. Acute swim stress elevated corticosterone levels, impairing spatial memory retention and reducing time spent in the target quadrant, suggesting that intense activation of stress pathways negatively affects cognitive function.
Avital et al. [15]	2006	Preclinical study	Mouse/Rat	Male Wistar rats, divided in 4 groups: MRA, GR antagonist, combined MR and GR antagonist, control	GR antagonist (RU38486/mifepristone), MRA (spironolactone), acute swim stress	Hippocampal plasticity (long term potentiation)	MR blockade impaired LTP, supporting that MR activation is essential for maintaining plasticity, particularly under stress.
Schwabe et al. [16]	2010	Preclinical study	Mouse/Rat	Male C57BL/6J mice, 12 weeks old	MRA (RU28318), restraint stress, corticosterone injection	Shift between spatial and stimulus–response (S-R) learning	Stress or corticosterone facilitated a switch from hippocampus-based spatial memory to caudate nucleus-based S-R memory, enhancing performance under stress. Spironolactone prevented the stress-induced shift to a habit-based memory system, leading to poorer spatial memory performance, suggesting that MR activation enables adaptive cognitive switching under stress, which supports cognitive resilience.
Douma et al. [17]	1999	Preclinical study	Mouse/Rat	42 male Wistar rats divided into 4 groups: sham group, ADX group, corticosterone replacement therapy, MRA group, GR antagonist group	MRA (RU28318), GR antagonist (RU38486), adrenalectomy	Muscarinic receptor expression in hippocampus	MRA increased muscarinic receptor expression in hippocampus regions CA1 and CA3 while GR antagonism had no effect, suggesting that blocking MR (as with spironolactone) disrupts cholinergic modulation in the hippocampus, potentially impairing cognitive function.
Maggio et al. [18]	2009	Preclinical study	Mouse/Rat	Male Wistar rats, 2–3 weeks old	Spironolactone (MRA), corticosterone, aldosterone, dexamethasone	Inhibitory synaptic currents in hippocampus	Spironolactone blocked corticosterone’s MR-mediated effects in the ventral hippocampus, reducing inhibitory signaling, likely increasing excitability in that region. Therefore, it disrupted the hippocampal balance necessary for optimal cognitive function under stress, suggesting it could potentially worsen cognitive decline.
Thai et al. [19]	2013	Preclinical study	Mouse/Rat	Male Sprague Dawley rats; groups: control, acute stress on set-shifting, acute stress on reversal	MRA (spironolactone) and GR antagonist (RU38486) with stress	Behavioral flexibility in learning	Spironolactone did not impact the facilitation of reversal learning by acute stress, suggesting that MR activation or blockade did not play a role in the observed cognitive effects.
Wang et al. [20]	2020	Preclinical study	Mouse/Rat	SHRs treated with eplerenone (*n* = 10), untreated SHRs (*n* = 10), control Wistar–Kyoto rats (*n* = 10)	MRA (eplerenone, 50 mg/kg/day)	Brain tissue changes in the hippocampus	Eplerenone reduced aldosterone levels, prevented cortical thinning, and reduced apoptosis in SHRs, suggesting that MR antagonism can protect against aldosterone-induced brain damage in hypertension.
Sakata et al. [5]	2012	Preclinical study	Mouse/Rat	Female KKAy (T2DM model) mice, WT controls	MRA (spironolactone, 50 mg/kg/day)	Cognitive function (Morris Water Maze, shuttle avoidance test)	Spironolactone improved cognitive function in female diabetic mice, suggesting MR antagonism mitigates cognitive decline associated with type 2 diabetes, especially in females, possibly through MR-mediated mechanisms.
Hira et al. [21]	2020	Preclinical study	Mouse/Rat	STZ-induced Alzheimer’s model, different treatment doses	MRA (eplerinone)	Cognitive performance, AChE inhibition	Eplerenone treatment improved memory and reduced AChE activity and neuroinflammation in an STZ-induced Alzheimer’s model, indicating potential cognitive protection through MR antagonism in Alzheimer’s disease models.
Martisova et al. [22]	2012	Preclinical study	Mouse/Rat, Cell Culture	Early-life stressed rats (maternal separation), SHSY-5Y neuroblastoma cells	MRA (spironolactone), corticosterone, JNK inhibitor	BACE expression, amyloid pathology	Spironolactone blocked corticosterone-induced increases in BACE and pJNK expression, suggesting MR antagonism may mitigate amyloidogenic processes, although direct effects on cognitive decline were not assessed.
Dorey et al. [23]	2011	Preclinical study	Mouse/Rat	Male C57BL/6 mice, intra-hippocampal injections	MRA (RU-28318), corticosterone	Memory retrieval in delayed alternation task	MR antagonism with RU-28318 blocked corticosterone-induced memory retrieval impairment, suggesting that MR activation in the dorsal hippocampus contributes to stress-related cognitive decline.
Solas et al. [24]	2013	Preclinical study	Mouse/Rat	Male C57BL/6 mice, 3 months old	MRA (spironolactone), chronic corticosterone treatment	Cognitive performance (novel object recognition)	Spironolactone reversed corticosterone-induced cognitive impairments and insulin resistance, suggesting MR antagonism may mitigate stress-related cognitive deficits.
Diaz-Otero et al. [25]	2018	Preclinical study	Mouse/Rat	Male C57Bl/6 mice with angiotensin II-induced hypertension	MRA (eplerenone)	Cognitive function (Barnes maze, novel object recognition)	Eplerenone prevented cognitive dysfunction in hypertensive mice by improving arteriole dilation and reducing microglia density, indicating MR antagonism may protect cognition by enhancing cerebrovascular function.
Chambers et al. [26]	2022	Preclinical study	Mouse/Rat	Male SHRSP rats with hypertension, Sprague Dawley controls	MRA (eplerenone)	Cognitive function (Y-maze), neuroinflammation markers	Eplerenone improved spatial memory and reduced neuroinflammation, indicating that MR antagonism may protect against cognitive decline and vascular damage in hypertension.
Pires et al. [27]	2018	Preclinical study	Mouse/Rat	Sprague Dawley rats, high-fat diet model	MRA (canrenoic acid, active spironolactone metabolite)	Cerebral artery remodeling, white matter injury	MR antagonism prevented artery remodeling and reduced white matter injury in obesity, suggesting a protective effect of MRAs like spironolactone against obesity-related cerebrovascular and cognitive decline risks.
Chen et al. [6]	2020	Preclinical study	Mouse/Rat	Swiss albino mice, amyloid-beta induced AD model	MRAs (spironolactone, eplerenone)	Cognitive performance (Morris water maze)	Spironolactone and eplerenone improved learning and memory in an Alzheimer’s model, likely by increasing BDNF, H2S, and Nrf2 levels, decreasing amyloid-beta, and reducing neuroinflammation in the brain.
Loscertales et al. [28]	1998	Preclinical study	Chick	Day-old chicks, passive avoidance task model	Nootropic (piracetam), MRA (RU28318), GR antagonist (RU38486)	Memory retention in passive avoidance task	The memory-enhancing effect of piracetam on long-term retention in a passive avoidance task was blocked when MR antagonist was administered.
Douma et al. [29]	1998	Preclinical study	Mouse/Rat	Adult male Wistar rats, divided into vehicle, MRA(RU28318), GR antagonist (RU38486), and combined MR + GR antagonist groups	MRA (RU28318), GR antagonist (RU38486)	Spatial learning in food-rewarded task	MR antagonism with RU28318 impaired reference memory and delayed working memory acquisition in spatial learning, suggesting MR activation is crucial for optimal memory function in spatial tasks.
Stephan et al. [30]	2022	Preclinical study	Mouse/Rat	Tcf4 transgenic mice with social defeat, treated with spironolactone, aripiprazole, or both	MRA (spironolactone)	Reversal learning, working memory	Spironolactone alone improved reversal learning in the schizophrenia model; however, co-treatment with aripiprazole reduced this benefit, highlighting spironolactone’s potential for specific cognitive improvements in schizophrenia.
Wang et al. [31]	2013	Preclinical study	Mouse/Rat	Male adult Wistar rats, early-life stress (maternal separation)	MRA (eplerenone)	LTP in hippocampus	MR antagonism blocked stress-induced LTP in the hippocampus, showing MR’s essential role in stress-related memory formation and indicating that MR activation supports cognitive resilience.
Dorey et al. [32]	2012	Preclinical study	Mouse/Rat	Male C57BL/6 mice, subjected to acute stress and treated with MRA (RU-28318) or GR antagonist (RU-38486)	MRA (RU-28318), GR antagonist (RU-38486)	Memory retrieval in delayed alternation task	MR antagonism prevented immediate (15 min post-stress) memory impairments, while GR involvement was delayed. Therefore, MR activation is critical for rapid memory retrieval under stress, with MR antagonism preventing early stress-induced memory deficits, while GR influences memory at later stages.
Mehdipour et al. [33]	2022	Preclinical study	Mouse/Rat	Male Sprague Dawley rats with Aβ injection to induce memory impairment	MRA (spironolactone at doses of 10, 25, and 50 mg/kg)	Memory performance, microglial activation (Iba1 protein)	Spironolactone reduced microglial activation (Iba1 levels) but did not improve memory impairment in β-amyloid-induced AD model, suggesting its anti-inflammatory effects without direct cognitive benefits.
Albernaz-Mariano et al. [34]	2022	Preclinical study	Mouse/Rat	Male Wistar rats, subjected to restraint stress and IL-mPFC spironolactone infusion	MRA (spironolactone)	Aversive memory extinction, corticosterone release	Infralimbic spironolactone blocked stress-induced corticosterone increase and prevented impairment in aversive memory extinction, suggesting MR antagonism supports adaptive extinction of stress-related memories.
Licata et al. [35]	2022	Preclinical study	Human cell lines, iPSC-derived motor neurons, Drosophila model	Cells and fly models expressing neurotoxic DPRs	MRA (spironolactone)	DPR levels and neuroprotection	Spironolactone decreased DPR levels by promoting autophagy-mediated degradation, suggesting potential protective effects against neurotoxicity in C9ALS/FTD, though its effects on cognitive decline specifically were not assessed.

**Figure 2:**
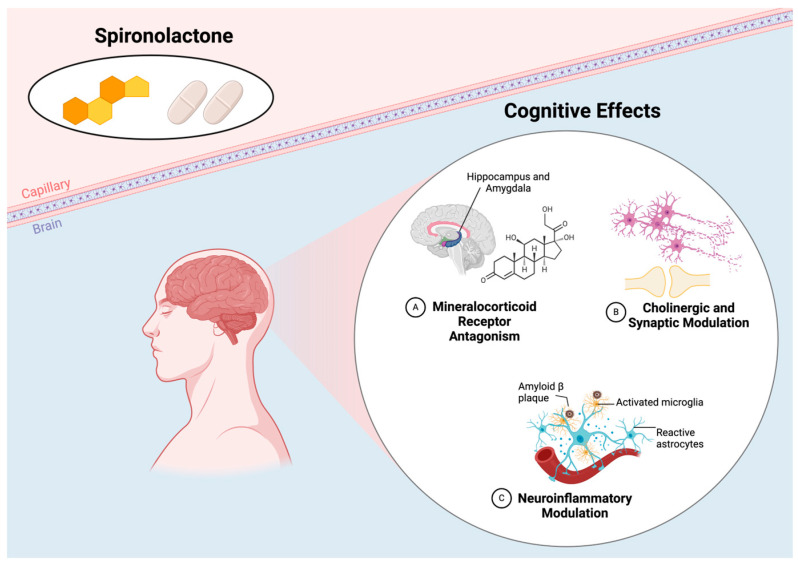
Mechanisms of MRAs in modulating cognitive outcomes. Created in https://BioRender.com (accessed on 12 December 2024) [36].

AD = Alzheimer’s disease; ADX = adrenalectomy; C9ALS/FTD = C9orf72-related amyotrophic lateral sclerosis/frontotemporal dementia; GR = glucocorticoid receptor; iPSC = induced pluripotent stem cell; LTP = long-term potentiation; MRA = mineralocorticoid receptor antagonist; SHRs = spontaneously hypertensive rats; STZ = streptozotocin; T2DM = type 2 diabetes mellitus. The underlying mechanisms are illustrated in Figure 2.

### 3.2. Clinical Studies

The clinical studies included diverse populations, such as healthy volunteers, individuals with psychiatric disorders, and patients with cardiovascular risk factors, and primarily examined the cognitive outcomes associated with the use of spironolactone and eplerenone. The main findings are reported in detail to emphasize the evidence derived from human research.

#### 3.2.1. Healthy Volunteers

Eight of the randomized trials were conducted on healthy volunteers. Table 2 provides a summary of the articles included. In all of these trials [37,38,39,40,41,42,43,44], spironolactone was administered to the participants of the active arm. Most studies were randomized controlled trials [37,38,39,40,41,42,43], while one study was a randomized, counter-balanced, double-blinded study [44]. Three studies were conducted by the same research group, Vogel et al., as part of a large-scale study investigating the effects of stress depending on mineralocorticoid receptor (MR) availability under the modulating effects of spironolactone [39,40,41]. Schwabe et al. authored two studies that were independent and not part of a larger-scale project [42,43]. We provide below a succinct summary of these eight randomized trials.

Cornelisse et al. assessed the effects of spironolactone on stress and observed that spironolactone improves long-term memory, which implies a potential stress-modulating impact on memory process. Also, spironolactone was associated with impaired selective memory in the absence of stress and had negative effects on working memory during stress. In all, spironolactone had variable effects on cognitive domains, with benefits on long-term memory but slightly adverse effects on selective and working memory [37].

In another randomized controlled trial, published by Otte et al. [38], the effects of spironolactone on cognitive function have been investigated with panic induction by administering cholecystokinin tetrapeptide (CCK-4). The results showed selective attention deficit, spatial/nonverbal memory decline, and diminished cognitive set shifting. There were no significant differences between the two groups regarding psychomotor activity, aspects of everyday memory, auditory verbal learning, or working memory [38].

Vogel et al. conducted three studies [39,40,41] exploring the effects of spironolactone on stress, cognition, and brain function. Stress was induced using a magnetic resonance imaging (MRI) compatible version of the Socially Evaluated Cold Pressor Test, while control groups underwent a non-stressful procedure. In the spatial memory task [39], no differences were found in recall errors between landmark-based and boundary-based objects or across stress and MR blockade conditions. Although spironolactone was associated with faster response there was no difference in accuracy. In addition, under stress, connectivity between the amygdala and striatum increased in the placebo group but remained unchanged in the spironolactone group. In the fear conditioning task [40], Vogel et al. used functional MRI to analyze brain activity in response to different stimuli to assess skin conductance response. In the absence of spironolactone, there was a stronger skin conductance response. Also, they highlighted that MR blockade may prevent stress-induced suppression of learning-related brain activity. In the emotional face-matching task [41], without spironolactone, stress resulted in enhanced connectivity between the centro-medial amygdala and caudate, extending to the putamen.

Schwabe et al. conducted two studies evaluating the effects of MR blockade with spironolactone on probabilistic classification learning (PCL), by analyzing different learning strategies used by the participants, and observing the engagement of the hippocampus and dorsal striatum using functional MRI, and inhibitory control [42,43]. In the PCL study [42], spironolactone administered before stress exposure significantly impaired classification learning performance, and the effect was not explained by cortisol levels alone. fMRI revealed activation in frontal, temporal, and parietal areas, with significant involvement of the hippocampus, caudate nucleus, and putamen during the PCL task. In the placebo group, increased caudate nucleus activation and negative correlation with hippocampal activation indicated a shift toward striatum-dependent learning under stress which had not been observed in the presence of spironolactone. Successful PCL performance was associated with stronger connectivity between the amygdala and hippocampus and between the amygdala and dorsal striatum [42]. In the second study [43], assessing inhibitory control using the “Go” and “Stop” test, MR blockade or stress had no influence on “Go” reaction times. The authors concluded that spironolactone might have a stabilizing effect on response inhibition under stress [43].

Young et al. [44] examined the effects of the glucocorticoid receptor (GR) mifepristone and MRA spironolactone on cognitive function and brain activity, showing specific patterns depending on emotional valence and treatment. GR antagonism enhanced activity in the amygdala during sad–neutral conditions and improved specific memory recall, whereas MR antagonism reduced the amygdala response in happy–neutral contrasts and enhanced recall of non-specific memories. Both antagonists diminished categorical memory recall. The whole-brain analysis revealed the regional neural differences between treatments that, according to treatment conditions, GR antagonism engaged regions implicated in the emotion and cognition of sad–neutral contrasts, and both antagonists reduced reward and emotional processing area activity for happy–neutral contrasts [44].

All studies consistently observed higher cortisol levels in the spironolactone group compared to placebo (*p* < 0.05 in all cases). This increase was attributed to spironolactone’s interference with the hypothalamic–pituitary–adrenal (HPA) axis, leading to reduced negative feedback to the anterior pituitary, increased release of ACTH, and consequently elevated cortisol levels [37,38,39,40,41,42,43,44,45].

**Table 2:** Clinical studies conducted on healthy volunteers.

Authors	Year	Study Type	Population	Exposure	Outcome	Main Findings
Cornelisse et al. [37]	2011	Randomized controlled trial	64 healthy young men divided into 4 groups: spironolactone-stress, spironolactone-no stress, no spironolactone-stress, no spironolactone-no stress	400 mg of spironolactone (MRA) versus placebo, combined with TSST or control task	Cognitive performance (selective attention, working memory, long-term memory), cortisol response, stress response	Spironolactone increased cortisol, impaired selective attention and working memory under stress, but improved long-term memory.
Otte et al. [38]	2007	Randomized placebo-controlled cross-over study	16 healthy young men divided into spironolactone and placebo groups, after one week washout inverted	300 mg of spironolactone (MRA) versus placebo, combined with a panic-inducing compound (CCK-4)	Effects on panic symptoms, cortisol/ACTH levels, and cognitive function (selective attention, visuospatial memory, set shifting)	Spironolactone impaired selective attention, visuospatial memory, and flexibility; increased baseline cortisol but had no effect on panic symptoms.
Vogel et al. [41]	2015	Randomized controlled trial	101 young healthy men, divided into 4 groups: stress-spironolactone, stress-placebo, control-spironolactone, control-placebo	Spironolactone (MRA) vs. placebo combined with stress induction	Effects on brain connectivity, specifically amygdala-striatal connectivity under stress	Spironolactone preserved cognitive control by preventing stress-induced amygdala–striatum connectivity shifts.
Schwabe et al. [42]	2013	Randomized controlled trial	Healthy participants, spironolactone vs. placebo	MRA (spironolactone, 300 mg)	Memory system engagement under stress	Spironolactone blocked stress-induced learning shifts to striatum, impairing cognitive flexibility.
Vogel et al. [39]	2017	Randomized controlled trial	Healthy male participants, divided into MR-blocked (spironolactone) and control groups with/without stress	MRA (spironolactone, 400 mg)	Spatial memory strategy (fMRI-based)	Spironolactone prevented stress-induced spatial memory shifts to striatum.
Schwabe et al. [43]	2013	Randomized controlled trial	Healthy adults, four groups: stress vs. control and spironolactone vs. placebo	MRA (spironolactone, 300 mg)	Response inhibition in stop-signal task	Spironolactone blocked stress-induced improvement in response inhibition.
Young et al. [44]	2016	Randomized, counter-balanced within-subjects design	10 healthy male participants	MRA (spironolactone, 600 mg), GR antagonist (mifepristone, 600 mg)	Autobiographical memory recall, amygdala response to emotional faces	Spironolactone impaired memory specificity and increased amygdala response to sad faces.
Vogel et al. [40]	2015	Randomized controlled trial	101 healthy men divided into 4 groups	400 mg of spironolactone (MRA) versus placebo, combined with stress (cold pressor test)	Effects on fear learning, memory consolidation (trace vs. delay conditioning), and neural activation	Spironolactone prevented cognitive decline under stress and blocked the stress-induced shift to amygdala-based learning.

ACTH = Adrenocorticotropic Hormone; MRA = mineralocorticoid receptor antagonist; TSST = Trier Social Stress Test.

#### 3.2.2. Individuals with Psychiatric Disorders

The effect of MRAs on cognition was assessed in patients with psychiatric comorbidities, including schizophrenia, major depressive disorder (MDD), and bipolar I disorder. Our review included five studies: three randomized controlled trials [46,47,48] and two longitudinal studies [49,50]. Table 3 provides a summary of the articles included.

An ongoing multicenter, randomized, placebo-controlled study, the SPIRO-TREAT trial, has been designed to evaluate the efficacy of spironolactone as an add-on treatment for cognitive impairment in schizophrenia [46]. The premise is based on preclinical findings showing that spironolactone improved schizophrenia-relevant behaviors in transgenic mouse models. The trial also investigates whether the cognitive effects of spironolactone correlate with improvements in general functioning and psychiatric symptoms. Long-term administration of medium to high doses of spironolactone may lead to side effects, such as an increased risk for cardiac arrhythmias due to elevated potassium levels, especially in patients concurrently taking antipsychotic drugs. Long-term spironolactone treatment might also increase the risk for gynecomastia, amenorrhea, and blood count changes [46].

Patients with major depressive disorder were assessed to evaluate changes in cortisol secretion and its association with cognitive improvement during treatment in a longitudinal study conducted by Hinkelmann et al. [49]. This study explored the cognitive effects of spironolactone as an add-on to escitalopram treatment. Although spironolactone had no overall significant effect on enhancing cognition, reductions in cortisol levels were associated with improvements in specific cognitive tasks. The study concluded that cognitive improvements were likely related to HPA axis normalization rather than a direct effect of spironolactone [49].

In a longitudinal study, Sukhapure et al. assessed the effects of individualized treatments, which included spironolactone, for polycystic ovarian syndrome (PCOS), on cognitive function, depression, and anxiety [50]. Results showed significant reductions in depression and anxiety symptoms among PCOS participants. However, spironolactone did not demonstrate specific cognitive function enhancement in PCOS patients, although it may lead to improvements in verbal learning, visual-spatial learning, psychomotor speed, reduced anxiety, and improved attention [50].

Another placebo-controlled study examined the effects of spironolactone on cognitive and emotional empathy in patients with MDD [47]. Results revealed that spironolactone reduced cognitive empathy in MDD patients, bringing their performance to the level of healthy controls. No significant effects were observed for emotional or for cognitive empathy in healthy participants. The Movie for the Assessment of Social Cognition (MASC) results showed no significant differences in cognitive empathy scores between groups or treatments [47].

In a study conducted by Zandifar et al. [48], the efficacy of spironolactone as an adjunctive treatment to sodium valproate was evaluated for cognitive function and mood symptoms in patients with bipolar I disorder during manic episodes. Spironolactone significantly improved cognitive function; however, no significant effects of spironolactone were observed on manic symptoms, sleep quality, appetite, or body mass index [48].

**Table 3:** Clinical studies conducted on individuals with psychiatric disorders.

Authors	Year	Study Type	Population	Exposure	Outcome	Main Findings
Hasan et al. [46]	2020	Randomized controlled trial	90 patients with schizophrenia	Spironolactone 100 mg, 200 mg, or placebo added to standard antipsychotic treatment	Changes in working memory, cognitive functions	Ongoing study evaluating spironolactone’s effect on working memory deficits in schizophrenia.
Hinklemann et al. [49]	2012	Longitudinal cohort study	102 participants: 52 patients diagnosed with depression, 50 healthy control individuals	SSRI treatment and an add-on treatment modulating the MRA (spironolactone)	Cognitive improvement and changes in cortisol secretion (salivary cortisol levels)	Cortisol reduction improved cognitive functions, but spironolactone had no significant effect.
Sukhapure et al. [50]	222	Longitudinal cohort study	73 participants with PCOS, 33 receiving drugs, 40 as control	MRA (spironolactone) and oral contraceptives	Changes in depression, anxiety symptoms, and cognitive function	Spironolactone improved depression and anxiety, however likely due to practice effects.
Wingenfeld et al. [47]	2016	Randomized controlled trial	MDD patients and healthy individuals treated with spironolactone or placebo	MRA (spironolactone, 300 mg)	Cognitive and emotional empathy levels	Spironolactone reduced cognitive empathy in MDD but did not affect emotional empathy.
Zandifar et al. [48]	2023	Randomized controlled trial	Bipolar I disorder patients in manic episode (*n* = 60)	MRA (spironolactone, 50 mg/day)	Cognitive performance (MMSE), mania severity, sleep quality	Spironolactone improved cognitive performance and mania severity.

MDD = major depressive disorder; MMSE = mini-mental state examination; MRA = mineralocorticoid receptor antagonist; PCOS = polycystic ovary syndrome; SSRI = selective serotonin reuptake inhibitor.

#### 3.2.3. Individuals with Cardiovascular Risk Factors

Table 4 provides a summary of the included articles; among the three papers evaluating the impact of MRA on cognitive function in patients with cardiovascular risk factors, two studies were randomized controlled trials [7,51], while the third one was an observational cohort study [52].

Yagi et al. [7] conducted a randomized controlled trial on patients with essential hypertension to investigate whether plasma aldosterone level is associated with cognitive impairment and whether MR blockade alleviates cognitive dysfunction in hypertensive patients. The investigators observed that MR blockade can improve cognitive function in hypertensive patients, most likely through reductions in plasma aldosterone concentration (PAC) and blood pressure. The study further reported that increased PAC is associated with CI, potentially due to aldosterone-induced microvascular circulation insufficiency and cerebrovascular remodeling in the hippocampus. According to the authors, hippocampal hypoperfusion and hippocampal sclerosis—both linked to CI—may be mitigated by MR blockade, which increases hippocampal blood flow. They also noted that aldosterone can cross the blood–brain barrier, and since MRs are abundantly expressed in the hippocampus, MR blockade may directly protect against aldosterone-induced cerebral damage and hippocampal dysfunction. Age, PAC, and a history of cerebral infarction were independent predictors for cognitive impairment [7].

A randomized controlled trial [51] has been conducted to evaluate the effect of MR antagonism on the hippocampal memory in obesity. Baseline hippocampal memory was negatively correlated with age and positively with female gender. In the spironolactone group during a period of six weeks, serum aldosterone increased, mean arterial pressure decreased and there was a significant improvement in the hippocampal memory as compared to the placebo group. There were no differences in the other types of memory between the groups indicating that the effects of MR blockade are targeted at hippocampal-dependent cognitive function [51].

In a cohort study by Hong et al. [52], patients with primary aldosteronism and no prior dementia, as well as patients with essential hypertension and no prior dementia, were included. The patients with primary aldosteronism were further divided into subgroups based on their treatment strategy: adrenalectomy (ADX) or MRA therapy. The study aimed to evaluate the risk of developing dementia, including Alzheimer’s disease, vascular dementia, or dementia from other causes. The incidence of all-cause dementia was higher in the primary aldosteronism group compared to the essential hypertension group. Among the primary aldosteronism patients, those treated with MRA had a significantly higher risk of all-cause dementia than the essential hypertension group. In contrast, the ADX subgroup showed no significant differences in dementia risk compared to the essential hypertension group. Additionally, the study observed that treatment with spironolactone in the MRA subgroup adversely affected glucose metabolism, as evident by a positive association between insulin resistance and post-treatment plasma aldosterone concentration, an effect not observed in the ADX subgroup [52].

**Table 4:** Clinical studies conducted on individuals with cardiovascular risk factors.

Authors	Year	Study Type	Population	Exposure	Outcome	Main Findings
Hong et al. [52]	2023	Observational Cohort Study	Patients with PA treated with MRA ADX	MRA vs. ADX	Dementia incidence in PA patients	MRA-treated PA patients had a higher risk of vascular dementia, compared to no increased risk for ADX patients.
Rotenstein et al. [51]	2015	Randomized Controlled Trial	Obese adults (BMI 30–45), randomized to spironolactone or placebo	MRA (spironolactone, 50 mg/day)	Paired-associate learning task	Spironolactone improved hippocampal memory in obese adults
Yagi et al. [7]	2011	Observational Study with MR Blocker Intervention	Hypertensive patients with high aldosterone levels	MR blockers (spironolactone, eplerenone)	MMSE	High aldosterone levels were linked to cognitive impairment. MR blockers improved MMSE scores.

ADX = adrenalectomy; BMI = body mass index; MMSE = mini-mental state examination; MR = mineralocorticoid receptor; MRA = mineralocorticoid receptor antagonist; PA = primary aldosteronism.

## 4. Discussion

This systematic review discuss evidence from diverse populations—healthy individuals, psychiatric patients, and those with cardiovascular risk factors—to investigate the cognitive effects of MRAs as a therapeutic agent and the potential link of these effects to cognitive function in cardiovascular patients. The findings indicate that though the cognitive effects of spironolactone vary across populations, its impact on the HPA axis, stress responses, and neurovascular health consistently appears as a central theme. These mechanisms are not only involved in the modulation of cognitive function but also reveal the therapeutic importance of MRAs in cardiovascular contexts.

### 4.1. Cognitive Effects in Healthy Individuals: Indirect Evidence for Cardiovascular Impact

**Figure 3:**
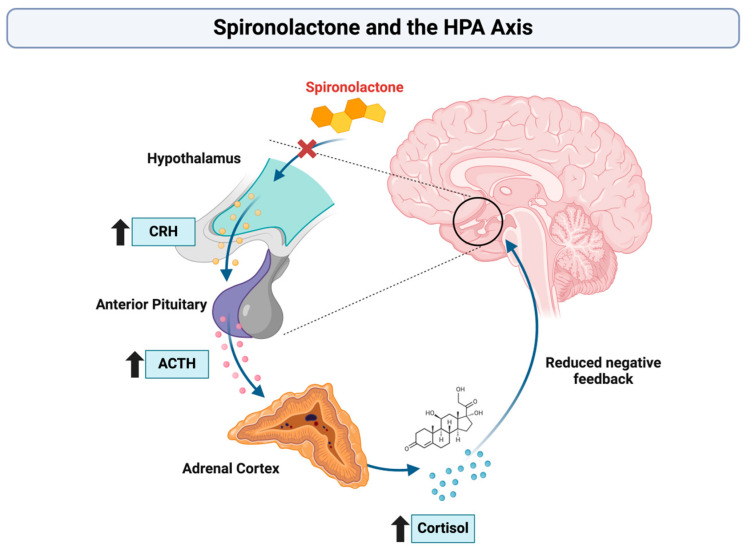
Influence of spironolactone on the hypothalamus–pituitary–adrenal axis. The upward arrows indicate an increase in CRH, ACTH, and cortisol levels. Created in https://BioRender.com (accessed on 17 December 2024) [36].

Healthy volunteer studies offer useful insights into spironolactone’s effects. Spironolactone was associated with improved long-term memory but impaired selective and working memory under stress [37]. This contrast matches the drug’s effects on cortisol and the brain’s stress response. Elevated cortisol observed in these studies demonstrates spironolactone’s disruption of MR-mediated feedback (Figure 3) [37,38,45]. Functional MRI studies revealed altered amygdala–striatum connectivity during stress, indicating that MR blockade affects brain regions essential for emotional and cognitive regulation [16,39,40,41,42,43,44]. These findings may have implications for vascular health, since stress-induced neurovascular dysfunction is a risk factor for hypertension and cerebrovascular events [53], suggesting potential pleiotropic benefits in patients with cardiovascular risk factors. However, it should be noted that the spironolactone doses that were administered to healthy subjects (300–600 mg daily) are an order of magnitude higher than the doses proven to provide benefits in patients with heart failure and resistant hypertension [54,55]. Further work is therefore needed to clarify the cognitive effects of “cardiovascular” doses of spironolactone.

### 4.2. Psychiatric Populations: Bridging Cognitive and Cardiovascular Outcomes

In psychiatric populations, spironolactone revealed mixed cognitive effects, which can be correlated with cardiovascular mechanisms. Spironolactone reduced cognitive empathy deficits in MDD patients, to the level of healthy controls, but did not improve emotional empathy [47]. Given the role of cortisol dysregulation in both depression and cardiovascular diseases, these findings highlight MRA’s capability to address mutual pathophysiological pathways [45]. In longitudinal studies, reductions in cortisol levels during spironolactone treatment correlated with specific cognitive improvements, such as attention and memory tasks [49]. This highlights the link between HPA axis regulation and vascular health, as hypercortisolism is a common feature of both depression and cardiovascular conditions [56]. In patients with bipolar I disorder, spironolactone improved cognitive function during manic episodes but did not significantly affect manic symptoms [48]. The lack of mood stabilization beside cognitive improvement implies that spironolactone’s effects may be regulated through systemic pathways, such as vascular or neuroendocrine modulation, rather than direct psychotropic effects.

### 4.3. Cardiovascular Populations: Direct Evidence of Cognitive Benefits

Spironolactone had the most consistent positive cognitive effects among patients with cardiovascular risk factors. Spironolactone significantly improved the mini-mental state examination, MMSE, in hypertensive patients, an effect likely mediated by reduced blood pressure and improved cerebral perfusion [7]. Although spironolactone improved some vascular markers, the spironolactone group experienced more cases of dementia than did the adrenalectomy group [52], suggesting that cognitive benefits of spironolactone may be associated with specific circumstances, particularly in cases with significant plasma aldosterone levels. Among obese subjects without other comorbidities, spironolactone improved hippocampal memory, the aspect of memory that is sensitive to vascular and inflammatory insults [51,57], suggesting that spironolactone may improve neurovascular health.

The evidence from healthy individuals, psychiatric patients, and cardiovascular populations suggests that spironolactone’s cognitive effects are linked with its systemic impact on cardiovascular health. Across populations, adjustment of cortisol levels and stress responses appears as a unifying mechanism. While elevated cortisol is beneficial for specific memory processes, it can impair vascular health and increase the risk of cardiovascular events which emphasizes the need for targeted dose therapeutic strategies. However, eplerenone may have more favorable effects on cortisol levels compared to spironolactone in chronic heart failure patients [58]. Improvements in cognitive function, particularly in cardiovascular populations, are likely mediated by enhanced cerebral perfusion and reduced vascular inflammation. This supports the role of MRAs as modulators of vascular health. The mixed outcomes in healthy and psychiatric populations may reflect (1) a lack of underlying cardiovascular pathology, and thus less pronounced systemic benefits of spironolactone and (2) “pharmacologic” doses of spironolactone (300–600 mg daily), which have been long abandoned in cardiovascular medicine in favor of “neurohormonal” doses (25–50 mg daily). By contrast, patients with cardiovascular risk factors show clear cognitive improvements, strengthening the hypothesis that the effects of spironolactone are most pronounced in conditions involving vascular or neuroendocrine dysfunction.

### 4.4. Study Limitations

The main limitations of our review are population heterogeneity, inconsistent assessment of cognitive function, short study duration, and lack of long-term data. Confounding factors were variable, including comorbidities and gender/age differences that render interpretation of finding more challenging. Data on dose–response associations and comparative data with eplerenone were limited and therefore the evidence on these areas remain inconclusive. Conflicting results regarding dementia risk and a number of underexplored mechanisms point out the need for standardized, long-term, population-specific research to clarify the cognitive effects of MRAs.

### 4.5. Implications for Future Research and Practice

Future research should prioritize investigating the shared pathways linking cognitive and cardiovascular effects, particularly focusing on the roles of the HPA axis and vascular health. Evaluating the long-term cognitive effects of MRAs in diverse populations with cardiovascular disease has the potential to reduce the adverse impact of cardiovascular disease and associated risk factors on cognition. Additionally, future studies should examine the cognitive effects of MRAs in patients who have both psychiatric disorders and cardiovascular risk factors, as these conditions frequently coexist and share mechanisms such as inflammation and neurovascular dysfunction. Understanding the role of MRAs in this comorbid population could enhance patient selection for MRA therapy and optimize cognitive benefits across complex clinical scenarios.

## 5. Conclusions

The findings of this systematic review highlight the importance of considering cardiovascular health when evaluating the effects of MRAs on cognition. While the benefits of MRAs are most evident in patients with cardiovascular risk factors, their impact extends through multiple physiological pathways, including the HPA axis and neurovascular function. MRAs appear to influence cognition primarily through cardiovascular-mediated mechanisms: improved vascular perfusion, reduced neurohormonal activation, and decreased systemic inflammation. While direct effects of MRAs on cognitive function through central mineralocorticoid receptors have been proposed, the evidence from this review suggests that the cognitive benefits are predominantly mediated through improvements in cardiovascular health. By targeting these cardiovascular-related pathways that contribute to cognitive decline, MRAs emerge as promising therapeutic agents, particularly for patients with concurrent cardiovascular risk factors. However, further research is warranted to fully elucidate any direct effects of MRAs on cognitive function and to identify the patient populations that might derive the greatest benefit.

## Data Availability

Not applicable.
